# Burnout and Interventions for Healthcare workers to cope during COVID-19

**DOI:** 10.1192/bjo.2021.404

**Published:** 2021-06-18

**Authors:** Harnek Kailey

**Affiliations:** Maidstone and Tunbridge Wells NHS Trust

## Abstract

**Aims:**

Healthcare workers are exposed to both physical and mental demands in the hospital environment; recently intensified by overstretching staff and resources during the current COVID-19 pandemic. Despite healthcare workers banding together, physician burnout is more prevalent than ever before due to emotional, physical and mental exhaustion. Firstly, this poster aims to expose the prevalence of burnout among healthcare workers during the COVID-19 pandemic. Secondly, to highlight the interventions and strategies to help minimise burnout among healthcare workers.

**Method:**

I will focus on reviewing clinical trials with a particular focus on healthcare workers affected by burnout within the COVID-19 pandemic timeframe. Therefore, narrowing my search to 20 trials within the past 12 months using the following restricted search criteria: ‘burnout’, ‘covid-19’ and ‘healthcare’. Furthermore, commenting on strategies and interventions to minimise burnout by stretching my criteria to interventions trialled within the last 24 months. This is due to limited data and trial evidence for burnout strategies within the last 12 months of the COVID-19 pandemic.

**Result:**

Burnout is on the rise among healthcare workers across the globe, 47% of healthcare staff are expressing an element of burnout worldwide. With growing concerns of healthcare staff developing long term mental health implications as a result of work-related stress. At present, one third of frontline staff are experiencing depression and distress; which must be addressed. Reviewing recent trials has highlighted a number of successful strategies for approaching burnout including: app technology, talking therapy, staff support and internet-based resources. App-related technology and web resources have shown to be particularly beneficial among recent trials, with limited participation and engagement for support groups/talking therapy.

**Conclusion:**

A significant rise in physician burnout and distress during COVID-19 has been noted in various trials. Interventions specifically related to burnout within COVID-19 are limited due to a low yield of completed trials; however, a couple of trials have found an improvement in COVID-19 related stress among healthcare workers using app-related technology. Internet based intervention is cheap, widely accessible and a non-judgemental method for seeking help, especially within a profession where burnout is heavily stigmatized.

